# Personal, Psychosocial and Environmental Factors Related to Sick Building Syndrome in Official Employees of Taiwan

**DOI:** 10.3390/ijerph15010007

**Published:** 2017-12-22

**Authors:** Chung-Yen Lu, Meng-Chuan Tsai, Chih-Hsin Muo, Yu-Hsien Kuo, Fung-Chang Sung, Chin-Ching Wu

**Affiliations:** 1 Department of Sport and Health Management, Da-Yeh University, Changhua 51591, Taiwan; chungyen@mail.dyu.edu.tw (C.-Y.L.); dykama@mail.dyu.edu.tw (M.-C.T.); 2 Management Office for Health Data, China Medical University Hospital, Taichung 40447, Taiwan; b8507006@gmail.com; 3 Department of Chinese Medicine, China Medical University Hospital, Taichung 40447, Taiwan; u100030033@cmu.edu.tw; 4 Department of Health Services Administration, China Medical University, Taichung 40402, Taiwan; 5 Department of Public Health, China Medical University, Taichung 40402, Taiwan

**Keywords:** sick building syndrome, building-related symptoms, psychosocial stress, work environment, indoor air

## Abstract

Sick building syndrome (SBS) is a combination of symptoms that can be attributed to exposure to specific building conditions. The present study recruited 389 participants aged 20–65 years from 87 offices of 16 institutions to examine if personal factors, work-related psychosocial stress, and work environments, were associated with five groups of SBS symptoms, including symptoms for eyes, upper respiratory tract, lower respiratory tract, skin, and non-specific systems. Indoor environmental conditions were monitored. Data were analyzed using multivariate logistic regression (MLR) analyses and were reported as adjusted Odds Ratios (aOR). SBS symptoms for eyes were associated with older age, sensitivity to tobacco, and low indoor air flow. Upper respiratory symptoms were related to smoking, low social support, longer work days, and dry air. High indoor air flow was associated with reduced upper respiratory symptoms (aOR = 0.29; 95% confidence interval (CI) = 0.13–0.67). Lower respiratory symptoms were associated with high work pressure, longer work hours, chemical exposure, migraine, and exposure to new interior painting. Recent interior painting exposure was associated with a high estimated relative risk of low respiratory symptoms (aOR = 20.6; 95% CI = 2.96–143). Smoking, longer work days, low indoor air flow, indoor dryness, and volatile organics exposure, were associated with other non-specified symptoms including headache, tiredness, difficulty concentrating, anger, and dizziness. In conclusion, there are various SBS symptoms associated with different personal characteristics, psychosocial, and environmental factors. Psychosocial factors had stronger relationships with lower respiratory symptoms than with other types of SBS symptoms. Good ventilation could reduce risk factors and may relieve SBS symptoms.

## 1. Introduction

Indoor environmental quality is important to health since most people in industrialized countries spend 80–90% of their life in indoor environments. This may lead to the risk of building-related problems for the general population [1,2]. In 1983, the World Health Organization (WHO) defined sick building syndrome (SBS) as certain medical symptoms, including headache, fatigue, and irritation in the upper respiratory tract, nose, throat, eyes, hands, and/or facial skin. These symptoms are associated with specific indoor and psychosocial work environments, and personal factors [3,4]. Studies on SBS have been carried out in various indoor environments. SBS risk has been found to be associated with female gender, allergies, personality traits, unbalanced psychosocial work environments, occupational stress, room temperature, humidity, building dampness, indoor air quality, outdoor air pollution, and meteorological conditions [5,6,7,8,9,10,11,12,13,14,15,16].

SBS is an important issue at work because it can lead to absenteeism and poor productivity among staff [17]. SBS is likely a combination of symptoms attributed to exposure to specific building conditions. Most recent studies on SBS have focused on indoor air pollution without considering job stressors and psychological work environments. With the rising awareness of work-related stress in developing countries, occupational stress has become a more serious issue, particularly in countries undergoing rapid economic development because this may increase the global burden of disease from occupational exposure [18]. In order to clarify the factors associated with SBS symptoms, the present study examined the role of personal factors, work-related psychosocial stress, and work environment associated with SBS, for office workers in high-rise buildings in Taiwan.

## 2. Materials and Methods

A letter was sent to employees working at 87 offices, randomly selected from 16 institutions located among high-rise buildings in Taipei city, inviting them to participate in the present study. The present study is a secondary analysis of data from previous studies, the details of which are presented elsewhere [9,10,13]. With informed consent, 389 people responded to our study (response rate 61.7%) and completed a self-report, confidential questionnaire collecting information on personal factors (gender, age, and smoking habits), psychosocial factors (work pressure, social support, workdays per week, working hours per day, tobacco sensitivity, chemical sensitivity, and migraine), environmental factors (sanitizing chemicals, recent interior painting, self-reported indoor air flow, and indoor dry air), and typical SBS symptoms experienced in the past month, specifically for eyes, upper and lower respiratory, skin, and non-specific complaints adapted from WHO Regional Office for Europe Report recommendations [3].

We measured air quality in each office, including carbon dioxide (CO_2_), total volatile organic compounds (TVOCs), and temperature (T), at 1.2 m height, far from any window or air-conditioner. CO_2_ and temperature were measured with Q-TRAK (IAQ Model 8551, TSI Inc., Shoreview, MN, USA) using electrochemical techniques with a detectable range of 0–5000 ppm and a thermistor with a range of 0–50 °C, respectively. The TVOCs were measured and calibrated for 102 volatile organic compound (VOC) categories with PID (PGM-7240, RAE System, Sunnyvale, CA, USA) using photo-ionization techniques with the range between 0 ppb and 199.9 ppm and an acceptable deviation of 20 ppb. Two point calibrations of CO_2_ (0 and 5000 ppm) and isobutylene gas (0 and 50,000 ppb) were performed before each sampling.

Indoor CO_2_ concentrations were classified into low (<800 ppm), medium (800–1000 ppm), or high (>1000 ppm) according to recommendations in the Mechanical Engineer’s Handbook (1916) [19] and the American Society of Heating, Refrigeration and Air Conditioning Engineers (ASHRAE) (1999) [20]. Indoor TVOC concentrations were also classified into low (<115 ppm), medium (115–406 ppm), or high (>406 ppm) groups. Indoor temperature classifications were based on the recommended indoor air quality value of 15–28 °C in Taiwan (2005) [21].

Participants were considered to suffer from building-related symptoms if they self-reported having one or more selected symptoms specified in the questionnaire while working in the office in the previous month. Symptoms must have been present for at least 1–3 days per week and improved or disappeared at the end of the working day or on weekends or vacations [3,9,22].

Data analysis initially measured the prevalence of specific SBS symptoms reported for 5 groups of complaints, including eyes (eye dryness and eye irritation), upper respiratory system (nose itching, runny nose, stuffy nose, sneezing and dry throat), lower respiratory system (difficulty breathing), skin (skin dryness), and non-specific building-related symptoms (headache, tiredness, difficulty concentrating, anger and dizziness). Distributions of these symptoms were presented according to potential risk factors, including personal, psychosocial, and environmental factors. Logistic regression analyses were used to calculated crude odds ratios (cOR) and 95% confidence intervals (CI) of the reported symptoms by the potential risk factors. A multivariate logistic regression (MLR) model was used to calculate the adjusted odds ratios (aOR) of the symptoms with all personal, psychosocial, and environmental factors, included in the model.

## 3. Results

Among the 389 participants, 77.1% were females and 59.1% were less than 50 years of age. Of the participants, 80% had never smoked and 8.5% had quit smoking. Table 1 displays the prevalence of symptoms for the different personal, psychosocial, and environmental factors. Among all participants, prevalence rates of building-related symptoms for eyes, upper respiratory system, lower respiratory system, skin, and non-specific symptoms, were 23.4%, 15.7%, 6.9%, 2.1% and 26.2%, respectively (Table 1).

Table 2 shows that smoking, psychosocial factors, and environmental factors were associated with SBS. The MLR models in Table 3 show that symptoms of eyes were associated with older age (aOR = 3.08; 95% CI = 1.18–8.07), tobacco sensitivity (aOR = 3.24; 95% CI = 1.42–7.36), and low indoor air flow inside the office (aOR = 2.95; 95% CI = 1.51–5.75).

Table 3 also shows that upper respiratory symptoms were related to dryness inside the office (aOR = 3.82; 95% CI = 1.77–8.25), current smoking (aOR = 4.84; 95% CI = 1.67–14.0), lower social support (aOR = 2.98; 95% CI = 1.45–6.13), and working more than 5 days a week (aOR = 3.72; 95% CI = 1.22–11.3). However, high indoor air flow was related to a reduced aOR of 0.29 (95% CI = 0.13–0.67). Lower respiratory symptoms were associated with work pressure (aOR = 7.17; 95% CI = 1.49–34.5), working more than 10 h a day (aOR = 5.16; 95% CI = 1.42–18.8), self-reported chemical sensitivity (aOR = 14.0; 95% CI = 2.03–96.2), and migraine (aOR = 6.44; 95% CI = 1.88–22.1). Recent painting inside the office was associated with lower respiratory symptoms with an aOR of 20.6 (95% CI = 2.96–143). Non-specific symptoms were found to relate to current smoking (OR = 4.34; 95% CI = 1.61–11.7), working more than 5 days a week (OR = 2.75; 95% CI = 1.10–6.90), self-reported low indoor air flow (OR = 2.88; 95% CI = 1.49–5.57), dry air inside the office (OR = 2.70; 95% CI = 1.43–5.62), and medium indoor TVOC concentration (OR = 5.03; 95% CI = 1.92–3.63).

## 4. Discussion

The present study showed that complaints related to eyes and non-specific symptoms were the most common building-related symptoms for both females and males. Smoking, working for more than 5 days a week, low indoor air flow, and dry air inside the office, were variables associated with both upper respiratory symptoms and non-specific symptoms. Smoking was positively associated with upper respiratory symptoms.

The high prevalence of building-related eye symptoms is concerning. Our data showed that older individuals were more likely to have complaints of eye symptoms. Van Tilborg et al. reported that dry eye symptoms had a negative impact on daily work activities [23]. They found that among 505 participants, up to 70% experienced eye symptoms that inhibited daily work activity. Chemicals in the air and low ventilation may lead to eye irritation. 

For work-related psychosocial factors, high work pressure, working for more than 10 h a day, and migraine, were positively associated with lower respiratory symptoms. Having low social support was significantly related to upper respiratory symptoms, while working for more than 5 days a week was associated with both upper respiratory and non-specific symptoms. Office workers with tobacco sensitivity were more likely to have eye symptoms than those without. Marmot et al. suggested that high job demands and low support were more likely to be associated with building-related symptoms than environmental conditions [24]. In the present study, it seems that reducing occupational stress can be beneficial in reducing upper and lower respiratory, and non-specific symptoms. However, our data also showed that chemical sensitivity was related to a significantly increased risk of lower respiratory symptoms. Respiratory symptoms induced by inhaled chemicals have been described as chemical sensitivity. This is a common problem and is frequently reported in population studies [25]. Stimuli-like chemicals can evoke sensory nerve potentials via the vanilloid receptor family on the C-fibres found in all parts of respiratory system. This can cause neurogenic inflammation and a cough [26,27]. The airway symptoms caused by chemicals may increase airway sensory nerve reactivity, like a cough reaction after capsaicin inhalation [28]. Our data showed that individuals with chemical sensitivity and migraine were at increased risk of lower respiratory but not upper respiratory symptoms. A US study using EPA nationwide data reported that migraines and headaches in office workers were associated with an uncomfortable indoor environment because of inadequate indoor air quality, illuminance, and noise [29].

Building-related symptoms were significantly associated with indoor environmental quality. A Malaysia study conducted among school students showed indoor concentrations of xylene, formaldehyde, NO_2_ and CO_2_ could be risk factors for ocular, throat, fatigue, and headache symptoms [30]. Nevertheless, indoor CO_2_ had no association with SBS in the present study. Higher indoor TVOC exposure seemed to be related to non-specific building-related symptoms; however, there was no dose–response effect. The VOC thresholds for sensory irritation varied greatly between species and were of a greater magnitude than their odor thresholds. The dose of VOC concentrations in offices may be so low that they cause sensory irritation in the eyes and respiratory tract on the basis of estimated thresholds for sensory irritation [31]. This might explain why self-reported tobacco and chemical sensitivity had a greater association with eye and airway symptoms than did indoor TVOC concentration in the present study.

People who worked in offices with dry air were more likely to have upper respiratory and non-specific symptoms than those without dryness. Recent interior painting in offices was the leading factor related to lower respiratory symptoms and difficulty breathing. Wieslander et al. found that occupational exposure to water-based paints could increase the prevalence of self-reported lower airway symptoms [32]. We also found that working in an office with low air flow was related to eye and non-specific symptoms, while strong air flow was associated with reduced upper respiratory symptoms. Further research with a larger sample size should be conducted to further investigate the risk of indoor air flow in offices.

Our findings must be interpreted with the awareness of limitations. Firstly, we used a cross-sectional survey which limited causal inferences and the self-report questionnaire was subject to recall bias. Secondly, our questionnaire did not collect information on the use of personal computers and cell phones, which might be related to eye symptoms. Thirdly, the self-reported assessments on health status, psychosocial, and several environmental conditions, may be biased or inaccurate. Fourthly, the prevalence of SBS might be underestimated because employees suffering from building-related symptoms might seek ways to relieve the discomfort. Fifthly, we assessed SBS-related indoor air quality for whole offices instead of individual offices, which limited our ability to assess exposure variability among the study population [33]. Finally, multiple comparisons were conducted to assess the associations between multiple factors and SBS. False findings could occur because of Type I error or misclassification from self-reported or under-reported data. This could explain the unexpected protective relation between smoking and lower respiratory symptoms. Hence, findings in this study were considered as exploratory rather than confirmatory. The sample size of the study might be too small to increase the likelihood of a Type II error, skewing the results and decreasing the power of the study.

## 5. Conclusions

In conclusion, employees in high-rise building offices are exposed to conditions that may increase the risk of various SBS symptoms. In addition to building-related environmental conditions, the risk factors could be self-generated, such as smoking and high work pressure. This study reveals that psychosocial factors are also important and significantly associated with SBS, particularly with regard to lower respiratory symptoms. It is likely that quitting smoking could effectively reduce upper respiratory and non-specific building-related symptoms in office staff. Improved indoor air quality and reduced work stress may prevent office workers from suffering from SBS. However, additional studies are needed with larger samples and more indoor air indicators, including carbon monoxide, carbon dioxide, nitrogen dioxide, ozone, reactive VOCs, humidity, and ventilation rate. 

## Figures and Tables

**Table 1:** Distributions of sick building syndrome symptoms by personal, psychosocial, and environmental factors, for eyes, upper respiratory system, lower respiratory system, skin, and non-specific symptoms (*n* = 389).

Variables		Eyes	Upper Respiratory	Lower Respiratory	Skin	Non-Specific
*n* (prevalence, %)		91 (23.4)	61 (15.7)	27 (6.90)	8 (2.10)	102 (26.2)
**Personal factors**						
Gender	Male	21 (23.6)	11 (12.4)	9 (10.1)	3 (3.40)	31 (34.8)
	Female	70 (23.3)	50 (16.7)	18 (6.00)	5 (1.70)	71 (23.7)
Age, years	<30	24 (23.8)	15 (14.9)	5 (5.00)	2 (2.00)	34 (33.7)
	30–39	35 (20.8)	37 (22.0)	11 (6.50)	4 (2.40)	51 (30.4)
	40–49	16 (25.8)	4 (6.50)	3 (4.80)	0	7 (11.3)
	≥50	16 (27.6)	5 (8.60)	8 (13.8)	2 (3.40)	10 (17.2)
Smoking habit	Never	72 (23.2)	41 (13.2)	20 (6.40)	5 (1.60)	67 (21.5)
	Former	10 (30.3)	2 (6.10)	6 (18.2)	0	13 (39.4)
	Current	9 (20.0)	18 (40.0)	1 (2.20)	3 (6.70)	22 (48.9)
**Psychosocial factors**						
High work pressure	No	76 (21.7)	56 (16.0)	18 (5.10)	8 (2.30)	85 (24.3)
	Yes	15 (38.5)	5 (12.8)	9 (23.1)	0	17 (43.6)
Low social support	No	53 (21.7)	26 (10.7)	14 (5.70)	7 (2.90)	56 (23.0)
	Yes	38 (26.2)	35 (24.1)	13 (9.00)	1 (0.70)	46 (31.7)
Work days per week	≤5	83 (24.2)	50 (14.6)	20 (5.80)	8 (2.30)	84 (24.5)
	>5	8 (17.4)	11 (23.9)	7 (15.2)	0	18 (39.1)
Work hours per day	≤10	69 (22.6)	45 (14.8)	16 (5.20)	8 (2.60)	80 (26.2)
	>10	22 (26.2)	16 (19.0)	11 (13.1)	0	22 (26.2)
Tobacco sensitivity	No	13 (10.7)	24 (19.7)	7 (5.70)	5 (4.10)	25 (20.5)
	Yes	78 (29.2)	37 (13.9)	20 (7.50)	3 (1.10)	77 (28.8)
Chemical sensitivity	No	20 (14.5)	25 (18.1)	4 (2.90)	4 (2.90)	29 (21.0)
	Yes	71 (28.3)	36 (14.3)	23 (9.20)	4 (1.60)	73 (29.1)
Migraine	No	75 (23.4)	46 (14.4)	16 (5.00)	8 (2.50)	83 (25.9)
	Yes	16 (23.2)	15 (21.7)	11 (15.9)	0	19 (27.5)
**Environmental factors**						
Sanitizing with chemicals	No	70 (26.0)	29 (10.8)	19 (7.10)	2 (0.70)	62 (23.0)
	Yes	21 (17.5)	32 (26.7)	8 (6.70)	6 (5.00)	40 (33.3)
Recent painting	No	87 (23.5)	60 (16.2)	21 (5.70)	8 (2.2)	97 (26.2)
	Yes	4 (21.1)	1 (5.3)	6 (31.6)	0	5 (26.3)
High indoor air flow	No	46 (18.4)	37 (14.8)	13 (5.20)	7 (2.80)	49 (19.6)
	Yes	45 (32.4)	24 (17.3)	14 (10.1)	1 (0.70)	53 (38.1)
Low indoor air flow	No	29 (15.0)	23 (11.9)	7 (3.60)	4 (2.1)	23 (11.9)
	Yes	62 (31.6)	38 (19.4)	20 (10.2)	4 (2.0)	79 (40.3)
Indoor dryness	No	45 (19.4)	18 (7.80)	13 (5.60)	3 (1.30)	36 (15.5)
	Yes	46 (29.3)	43 (27.4)	14 (8.90)	5 (3.20)	66 (42.0)
CO_2_, ppm	<800	33 (24.3)	11 (8.10)	7 (5.10)	0	21 (15.4)
	800–1000	24 (23.1)	18 (17.3)	13 (12.5)	0	25 (24.0)
	>1000	34 (22.8)	32 (21.5)	7 (4.70)	8 (5.40)	56 (37.6)
TVOCs, ppb	<115	34 (25.6)	20 (15.0)	6 (4.5)	0	16 (12.0)
	115–406	28 (22.2)	13 (10.3)	16 (12.7)	0	39 (31.0)
	>406	29 (22.3)	28 (21.5)	5 (3.80)	8 (6.20)	47 (36.2)
Temperature, °C	15–28	87 (23.2)	60 (16.0)	21 (5.60)	8 (2.10)	96 (25.6)
	<15 or >28	4 (28.6)	1 (7.10)	6 (42.9)	0	6 (42.9)

**Table 2:** Crude odds ratios of sick building syndrome symptoms in relation to personal, psychosocial, and environmental factors (*n* = 389).

Variables		Eye	Upper Respiratory	Lower Respiratory	Non-Specific
**Personal factors**					
Gender	Male	1.00	1.00	1.00	1.00
	Female	0.99 (0.56–1.72)	1.42 (0.70–2.86)	0.57 (0.25–1.31)	0.58 (0.35–0.97)
Age, years	<30	1.00	1.00	1.00	1.00
	30–39	0.84 (0.47–1.52)	1.62 (0.84–3.13)	1.35 (0.45–3.99)	0.86 (0.51–1.46)
	40–49	1.12 (0.54–2.32)	0.40 (0.13–1.25)	0.98 (0.23–4.24)	0.25 (0.10–0.61)
	≥50	1.22 (0.59–2.55)	0.54 (0.19–1.57)	3.07 (0.96–9.88)	0.41 (0.19–0.91)
Smoking habit	Never	1.00	1.00	1.00	1.00
	Former	1.44 (0.66–3.17)	0.43 (0.10–1.84)	3.23 (1.20–8.74)	2.37 (1.12–5.01)
	Current	0.83 (0.38–1.80)	4.39 (2.22–8.67)	0.33 (0.04–2.53)	3.48 (1.83–6.63)
**Psychosocial factors**					
High work pressure	No	1.00	1.00	1.00	1.00
	Yes	2.25 (1.13–4.51)	0.77 (0.29–2.06)	5.53 (2.29–13.4)	2.41 (1.22–4.75)
Low social support	No	1.00	1.00	1.00	1.00
	Yes	1.28 (0.79–2.07)	2.67 (1.53–4.66)	1.62 (0.74–3.55)	1.56 (0.99–2.47)
Work days per week	≤5	1.00	1.00	1.00	1.00
	>5	0.66 (0.30–1.47)	1.84 (0.88–3.86)	2.90 (1.15–7.29)	1.98 (1.04–3.76)
Work hours per day	≤10	1.00	1.00	1.00	1.00
	>10	1.21 (0.70–2.12)	1.36 (0.72–2.55)	2.72 (1.21–6.12)	1.00 (0.58–1.73)
Tobacco sensitivity	No	1.00	1.00	1.00	1.00
	Yes	3.46 (1.84–6.52)	0.66 (0.37–1.16)	1.33 (0.55–3.24)	1.57 (0.94–2.63)
Chemical sensitivity	No	1.00	1.00	1.00	1.00
	Yes	2.33 (1.35–4.03)	0.76 (0.43–1.32)	3.38 (1.14–9.98)	1.54 (0.94–2.52)
Migraine	No	1.00	1.00	1.00	1.00
	Yes	0.99 (0.53–1.83)	1.66 (0.86–3.18)	3.60 (1.59–8.16)	1.09 (0.61–1.95)
**Environmental factors**					
Sanitizing with chemicals	No	1.00	1.00	1.00	1.00
	Yes	0.60 (0.35–1.04)	3.01 (1.72–5.26)	0.94 (0.40–2.21)	1.67 (1.04–2.68)
Recent painting	No	1.00	1.00	1.00	1.00
	Yes	0.87 (0.28–2.68)	0.29 (0.04–2.19)	7.67 (2.65–22.2)	1.01 (0.35–2.86)
High indoor air flow	No	1.00	1.00	1.00	1.00
	Yes	2.12 (1.32–3.43)	1.20 (0.69–2.11)	2.04 (0.93–4.48)	2.53 (1.59–4.02)
Low indoor air flow	No	1.00	1.00	1.00	1.00
	Yes	2.62 (1.59–4.30)	1.78 (1.01–3.12)	3.02 (1.25–7.32)	4.99 (2.97–8.40)
Indoor dryness	No	1.00	1.00	1.00	1.00
	Yes	1.72 (1.07–2.77)	4.48 (2.47–8.13)	1.65 (0.75–3.61)	3.95 (2.45–6.36)
CO_2_, ppm	<800	1.00	1.00	1.00	1.00
	800–1000	0.94 (0.51–1.71)	2.38 (1.07–5.29)	2.63 (1.01–6.86)	1.73 (0.91–3.31)
	>1000	0.92 (0.53–1.60)	3.11 (1.50–6.45)	0.91 (0.31–2.66)	3.30 (1.86–5.84)
TVOCs, ppb	<115	1.00	1.00	1.00	1.00
	115–406	0.83 (0.47–1.48)	0.65 (0.31–1.37)	3.08 (1.16–8.14)	3.28 (1.72–6.25)
	>406	0.84 (0.47–1.48)	1.55 (0.82–2.92)	0.85 (0.25–2.85)	4.14 (2.19–7.80)
Temperature, °C	15–28	1.00	1.00	1.00	1.00
	<15 or >28	1.32 (0.41–4.33)	0.40 (0.05–3.15)	12.6 (4.02–39.8)	2.18 (0.74–6.44)

**Table 3:** Adjusted odds ratios of sick building syndrome symptoms in relation to personal, psychosocial, and environmental factors (*n* = 389).

Variables		Eye	Upper Respiratory	Lower Respiratory	Non-Specific
**Personal factors**					
Gender	Male	1.00	1.00	1.00	1.00
	Female	0.70 (0.32–1.58)	3.84 (1.21–12.2)	0.29 (0.05–1.71)	0.86 (0.38–1.95)
Age, years	<30	1.00	1.00	1.00	1.00
	30–39	0.97 (0.49–1.89)	1.10 (0.48–2.51)	1.90 (0.45–8.02)	0.77 (0.39–1.55)
	40–49	2.22 (0.92–5.37)	0.30 (0.08–1.15)	4.14 (0.59–29.1)	0.27 (0.09–0.79)
	≥50	3.08 (1.18–8.07)	0.73 (0.20–2.70)	4.17 (0.53–32.9)	0.95 (0.33–2.77)
Smoking habit	Never	1.00	1.00	1.00	1.00
	Former	0.62 (0.20–1.93)	0.34 (0.06–1.85)	0.37 (0.05–2.94)	1.30 (0.43–3.89)
	Current	1.33 (0.50–3.55)	4.84 (1.67–14.0)	0.07 (0.01–0.95)	4.34 (1.61–11.7)
**Psychosocial factors**					
High work pressure	No	1.00	1.00	1.00	1.00
	Yes	1.67 (0.71–3.93)	0.68 (0.20–2.34)	7.17 (1.49–34.5)	2.06 (0.81–5.22)
Low social support	No	1.00	1.00	1.00	1.00
	Yes	1.23 (0.70–2.18)	2.98 (1.45–6.13)	0.61 (0.19–1.94)	1.32 (0.71–2.44)
Work days per week	≤5	1.00	1.00	1.00	1.00
	>5	0.64 (0.24–1.68)	3.72 (1.22–11.3)	3.65 (0.94–14.2)	2.75 (1.10–6.90)
Work hours per day	≤10	1.00	1.00	1.00	1.00
	>10	1.12 (0.57–2.21)	1.00 (0.41–2.44)	5.16 (1.42–18.8)	0.90 (0.43–1.90)
Tobacco sensitivity	No	1.00	1.00	1.00	1.00
	Yes	3.24 (1.42–7.36)	1.40 (0.56–3.50)	0.4 (0.07–1.74)	1.93 (0.85–4.38)
Chemical sensitivity	No	1.00	1.00	1.00	1.00
	Yes	1.60 (0.81–3.17)	0.64 (029–1.40)	14.0 (2.03–96.2)	1.87 (0.93–3.94)
Migraine	No	1.00	1.00	1.00	1.00
	Yes	0.54 (0.26–1.14)	1.63 (0.66–4.03)	6.44 (1.88–22.1)	0.91 (0.42–1.98)
**Environmental factors**					
Sanitizing with chemicals	No	1.00	1.00	1.00	1.00
	Yes	0.75 (0.40–1.44)	1.93 (0.93–3.98)	1.49 (0.39–5.67)	1.72 (0.90–3.31)
Recent painting	No	1.00	1.00	1.00	1.00
	Yes	1.16 (0.31–4.29)	0.16 (0.02–1.76)	20.6 (2.96–143)	0.77 (0.19–3.14)
High indoor air flow	No	1.00	1.00	1.00	1.00
	Yes	1.75 (0.92–3.33)	0.29 (0.13–0.67)	1.74 (0.43–7.09)	1.10 (0.58–2.07)
Low indoor air flow	No	1.00	1.00	1.00	1.00
	Yes	2.95 (1.51–5.75)	1.31 (0.62–2.79)	2.53 (0.55–11.6)	2.88 (1.49–5.57)
Indoor dryness	No	1.00	1.00	1.00	1.00
	Yes	1.11 (0.61–2.03)	3.82 (1.77–8.25)	1.36 (0.37–4.97)	2.70 (1.43–5.62)
CO_2_, ppm	<800	1.00	1.00	1.00	1.00
	800–1000	1.05 (0.49–2.25)	1.25 (0.44–3.69)	3.83 (0.72–20.4)	1.21 (0.48–3.06)
	>1000	0.87 (0.23–3.27)	3.11 (0.58–16.7)	8.26 (0.73–93.0)	1.55 (0.43–5.62)
TVOCs, ppb	<115	1.00	1.00	1.00	1.00
	115–406	0.64 (0.29–1.41)	0.90 (0.31–2.59)	3.20 (0.62–16.7)	5.03 (1.92–13.1)
	>406	0.93 (0.22–3.94)	0.63 (0.11–3.75)	0.66 (0.05–9.00)	4.39 (0.98–19.6)
Temperature, °C	15–28	1.00	1.00	1.00	1.00
	<15 or >28	0.52 (0.09–3.17)	1.22 (0.10–15.6)	7.65 (0.60–97.6)	0.54 (0.08–3.63)
